# *Ulva lactuca* Extract Increases the Growth of *Lactuca sativa* var. *longifolia* by Regulating Physiological States and Soil Microorganisms

**DOI:** 10.3390/biology15211936

**Published:** 2026-10-25

**Authors:** Dongshun Fang, Mengying Yang, Qiyun Xie, Zihan Zhang, Mingjiang Wu, Haibin Tong, Binbin Chen

**Affiliations:** 1 National and Local Joint Engineering Research Center of Ecological Treatment Technology for Urban Water Pollution, Wenzhou University, Wenzhou 325035, China; fds15167974512@163.com (D.F.); wmj@wzu.edu.cn (M.W.); tonghb@wzu.edu.cn (H.T.); 2 College of Life and Environmental Science, Wenzhou University, Wenzhou 325035, China; 15258697816@163.com (M.Y.); x.qiyun@outlook.com (Q.X.); 13738771892@163.com (Z.Z.); 3 Zhejiang Provincial Key Laboratory of Water Ecological Environment Treatment and Resource Protection, Wenzhou University, Wenzhou 325035, China

**Keywords:** seaweed aquaculture, agricultural sustainable development, epiphytic algae, resource utilization, rhizosphere microbiota, soil amelioration

## Abstract

In the context of carbon peaking and carbon neutrality goals, large-scale macroalgal cultivation provides a sustainable source of marine biomass, whereas epiphytic algae, although often regarded as a constraint in seaweed farming, remain an underutilized resource. In this study, biomass of the epiphytic green alga *U. lactuca* was collected from the surface of cultivated *S. fusiforme*, processed into a concentrated aqueous extract, and applied to lettuce (*Lactuca sativa* var. *longifolia*) at dilution ratios of 1:600, 1:400, and 1:200. The results showed that the algal extract affected lettuce growth and quality in a concentration-dependent manner. The 1:600 treatment increased soluble sugar, soluble protein, and vitamin C contents by 134.25%, 31.65%, and 20.62%, respectively, while the 1:400 treatment produced the greatest increase in shoot biomass. The extract was also associated with changes in selected soil properties, including pH. High-throughput sequencing indicated that the treatment altered the composition of the rhizosphere-associated bacterial community, including shifts in the relative abundance of several taxa. Overall, these results suggest that appropriate application of epiphytic algal extract may serve as a promising improvement to lettuce growth and quality, with potential value for sustainable agricultural production.

## Simple Summary

Seaweed farming is increasingly recognized as a sustainable means of food production and a pathway for carbon emission reduction. However, the epiphytic algae that proliferate on cultivated macroalgae are usually regarded as waste or even as a constraint on production. Here we tested whether these epiphytes can be valorized. We harvested the epiphytic green algae *Ulva lactuca* from farmed *Sargassum fusiforme*, prepared an aqueous seaweed extract, and applied it to lettuce at three concentrations. The lowest concentration enhanced the nutritional quality of lettuce, whereas a moderately higher concentration promoted plant growth. The extract also modified soil properties and increased both the abundance and diversity of beneficial microorganisms in the lettuce rhizosphere. These results indicate that epiphytic algae generated during seaweed farming can be converted into a value-added product that supports more productive and sustainable crop cultivation, offering a practical route for transforming seaweed-farming by-products into agricultural resources.

## 1. Introduction

Macroalgae possess substantial economic value, and their large-scale cultivation had been widely adopted worldwide. They are rich in proteins, carbohydrates, fatty acids, minerals, and vitamins [1], demonstrating high nutritional value. Notably, through the assimilation of nitrogen, phosphorus, and carbon dioxide from seawater, macroalgae can mitigate eutrophication and enhance marine carbon sequestration [2]. In the context of the dual-carbon strategy, macroalgae are emerging as a key source of ocean-derived green food and a critical component of sustainable development.

During the cultivation of massive brown seaweeds, epiphytic fouling by opportunistic algae is a nearly ubiquitous phenomenon in marine ecosystems. *S. fusiforme*, one of the principal seaweed species farmed in China, is particularly susceptible to such colonization, with the foliose green algae *U. lactuca* being the dominant epibiont [3]. During the initial sampling investigation in the laboratory, a total of 17 species of epiphytic algae were recorded throughout the entire cultivation cycle, including 6 green algae, 9 red algae, and 2 brown algae. Among them, 8 species were previously unrecorded epiphytic macroalgae on *S. fusiforme*. During the growth of *S. fusiforme*, *U. lactuca* was the dominant epiphytic alga and was found throughout the entire cultivation cycle. Especially in the early stage of cultivation, its biomass ranged from 156.00 to 512.33 g·m^−2^ (Table S1). Due to its thin thallus and exceptionally high surface-to-volume ratio, *U. lactuca* achieves superior photosynthetic efficiency; once established on *S. fusiforme*, it markedly suppresses host growth and photosynthetic performance. Under high-density farming conditions, although both algae exhibit reduced performance, *U. lactuca* maintains relatively higher growth and photosynthetic rates, thereby intensifying competitive pressure and further compromising the vegetative and reproductive output of the host seaweed [4]. Moreover, epiphytic algae typically exhibit rapid growth and high reproductive output [5], enabling them to form dense canopies on host surfaces that drive down the standing biomass of the cultivated seaweed. Many epibionts also exude allelochemicals that exerted negative chemotropic effects, suppressing host growth, impairing fertility, and even causing mortality [6]. The fouling community might further attract herbivores, intensifying damage to the crop [7,8]. At that time, epiphyte control relies primarily on manual removal or chemical treatments; citric acid, for example, has been used to eliminate *U. lactuca* from *S. fusiforme* [9]. However, rather than being treated solely as pests, these algae can be regarded as valuable germplasm resources and potentially valorized, offering a sustainable alternative to simple eradication.

Studies had demonstrated that seaweed extracts are a rich source of biostimulants that directly enhance crop growth. Polysaccharides, phytohormones, polyunsaturated fatty acids, compatible solutes, and trace elements present in these extracts collectively improve plant performance [6,10]. It has been reported in the literature that substances extracted from *Laminaria* spp. contain amino acids, mineral nutrients, and other bioactive compounds, including macronutrients such as potassium and phosphorus. (Table S2) [11,12]. The positive regulation exerted by seaweed extracts on plants includes increasing leaf chlorophyll content, soluble protein levels, and endogenous hormone concentrations [13], reducing disease incidence and enhancing resistance to pathogens [14], and acting as elicitors that up-regulate key genes involved in flowering and stress tolerance [15,16]. Moreover, the combined application of seaweed extracts with conventional fertilizers has been shown to increase biomass while lowering fertilizer input [17], thereby reducing agricultural reliance on synthetic nutrients and improving soil health [18]. *Lactuca sativa* var. *longifolia* was an annual or biennial herb belonging to the Asteraceae family. It is rich in vitamins, minerals, dietary fiber, flavonoids, and other bioactive compounds [19] and demonstrates auxiliary protective effects against cardiovascular disorders, oxidative stress, and immune decline. As a result, it ranks among the most widely cultivated leafy vegetables in China.

This study aimed to elucidate the regulatory effects of an *U. lactuca*-based extract on the growth and quality of lettuce (*L. sativa* var. *longifolia*). We hypothesized that foliar application of the extract would enhance lettuce growth and quality and would modulate the rhizosphere microbiome in a concentration-dependent manner. Although numerous studies have investigated *Ulva*-based extracts or ulvan as plant biostimulants, fewer studies have assessed the combined responses of crop performance, nutritional quality, and rhizosphere microbial communities to *U. lactuca*-derived extracts under a defined concentration gradient. By integrating these aspects, the present study provides new insights into the agricultural potential of *U. lactuca* biomass and its valorization as a sustainable biostimulant resource.

## 2. Materials and Methods

### 2.1. Experimental Materials and Design

Epiphytic green algal biomass was harvested from the culture rafts and thalli of cultivated *S. fusiforme* in Dongtou District, Wenzhou, Zhejiang Province. Based on previous laboratory findings and morphological observations, *U. lactuca* was selected as the target species [3]. Samples were sealed in polyethylene bags, transported to the laboratory, rinsed with sterile artificial seawater to remove visible epibionts and debris, and lyophilized to constant weight. The dried material was ground into a fine powder with a laboratory mill, passed through an 80-mesh sieve, packed in airtight bags, and stored at −20 °C until extraction.

For extraction, the dried algal powder was suspended in distilled water at a ratio of 1:20 (*w*/*v*), sonicated at 500 W for 40 min, and then incubated in a 60 °C water bath for 30 min. After cooling, the mixture was centrifuged at 9000 rpm for 10 min at 4 °C and used within 7 days after preparation.

Because the whole experiment lasted more than 45 days, the extract was not prepared once for the entire experimental period; instead, fresh stock extract was prepared in multiple batches as needed during the experiment. Each batch was prepared from the same stored powder using the identical extraction procedure, including the same powder-to-water ratio, sonication conditions, water-bath temperature and duration, centrifugation settings, and storage conditions, in order to minimize batch-to-batch variation. The application method of the extract solution is to uniformly spray it onto the surface of the lettuce leaves and the soil around the roots.

The extract used in this study was not chemically characterized at the time of experimentation. Therefore, any discussion regarding the potential roles of mineral nutrients or bioactive compounds in the extract is based on published reports on algal extracts and should be considered tentative.

Seeds of *L. sativa* var. *longifolia* were purchased from Shouhe Seed Co. (Weifang City, Shandong, China). After >50% of the seeds had germinated (radicle emergence), they were sown in a potting mix of loam:coconut coir (3:1, *v*/*v*) filled into plastic pots (15.5 cm top diameter, 12 cm height) at one plant per pot. Plants were raised in a growth chamber under a 16 h light/8 h dark photoperiod (200 µmol photons m^−2^ s^−1^), 22 °C and 70% relative humidity. Four treatments were arranged in a completely randomized design (*n* = 5): (i) high-nitrogen fertilization only (control), and high-nitrogen fertilization plus seaweed extract diluted (ii) 1:200, (iii) 1:400, or (iv) 1:600. The high-nitrogen fertilizer used was Stanley compound fertilizer, applied at 2 g per plant, with the same application frequency as the seaweed extract, i.e., on days 21, 31, and 39 after sowing. The seaweed extract was sprayed at 350 mL per application, and the control group was sprayed with an equal volume of clean water.

### 2.2. Assessment of Photosynthetic Characteristics

Net photosynthetic rate (Pn), stomatal conductance (Gs) and transpiration rate (Tr) were measured with a LI-6400 portable photosynthesis system (USA) following the protocol of Kumar et al. [20].

For photosynthetic pigment determination, fresh leaves (0.1 g) were ground in liquid nitrogen, homogenized in 10 mL of 95% ethanol, and incubated in darkness for 20 min. The extract was centrifuged at 6000 rpm for 5 min at 4 °C, and absorbances of the supernatant were read at 665, 649 and 470 nm. Chlorophyll a, chlorophyll b and total carotenoid contents (mg·g^−1^ fresh weight) were calculated using the equations of Lichtenthaler [21] with minor modifications.

### 2.3. Quantitative Analysis of Biochemical Components

Determination of soluble protein (SP), fresh leaf tissue (0.1 g) was ground in liquid nitrogen with 1 mL ice-cold PBS (pH 6.8). The mortar was rinsed with a further 7 mL PBS, and the combined homogenate was transferred to a 10 mL tube and centrifuged at 4000 rpm for 10 min. An aliquot (1 mL) of the supernatant was mixed with 5 mL Coomassie Brilliant Blue G-250 solution, incubated for 3 min, and the absorbance was read at 595 nm. SP content (mg·g^-1^ fresh weight) was calculated from a bovine serum albumin standard curve according to Bradford [22].

Determination of soluble carbohydrates (SC), soluble carbohydrates were quantified following Ershadi et al. [23] with minor modifications. Fresh leaf tissue (0.1 g) was homogenized in 1 mL distilled water, 1 mL MgCO_3_ slurry was added, and the volume was brought to 10 mL with distilled water. The extract was centrifuged at 10,000 rpm for 10 min. An aliquot (1 mL) of the supernatant, diluted 10-fold, was mixed with 2 mL distilled water and 10 mL anthrone reagent. After degassing, the mixture was heated in a boiling-water bath for 5 min, cooled immediately on ice, and brought to room temperature. Absorbance was read at 620 nm, and SC content (mg·g^−1^ fresh weight) was calculated against a glucose standard curve.

Ash content was determined by the method of Tian et al. [3] with minor modifications. Approximately 1 g of oven-dried, finely ground sample was weighed into a pre-ignited porcelain crucible and ashed in a muffle furnace at 550 °C for 6 h until a constant white/grey ash was obtained. After cooling in a desiccator, the crucible was re-weighed. Ash content (%) was calculated as:

Ash (%) = (weight of ash/weight of sample) × 100.

Vitamin C (ascorbic acid) was quantified by a molybdenum-blue colorimetric assay adapted from Wang et al. [24]. Fresh leaf tissue (0.5 g) was rapidly ground in liquid nitrogen and immediately mixed with 5 mL oxalic acid-EDTA solution, 0.5 mL metaphosphoric–acetic acid solution and 1 mL 5% (*w*/*v*) H_2_SO_4_. After addition of 2 mL 5% ammonium molybdate, the mixture was diluted to 25 mL with distilled water, homogenized, and centrifuged at 4000 rpm (20 °C, 10 min). The supernatant was allowed to stand at room temperature for 5 min and its absorbance was read at 760 nm. Ascorbic acid content (mg·100 g^−1^ fresh weight) was calculated from an ascorbate standard curve.

### 2.4. Determination of Total Flavonoids and Total Phenolics

Total flavonoids were determined following Saeed et al. [25] with minor modifications. Leaf powder (0.2 g) was extracted with 5 mL 70% ethanol under sonication for 30 min. An aliquot (0.2 mL) of the supernatant was transferred to a 10 mL tube, mixed with 0.15 mL 5% NaNO_2_ and left for 6 min; 0.15 mL 10% Al(NO_3_)_3_ was added, and after 6 min, 2.0 mL 4% NaOH was added. The volume was adjusted to 5 mL with distilled water, mixed, and allowed to stand for 3 min. Absorbance was read at 510 nm. Total flavonoid content was expressed as mg rutin equivalents g^−1^ dry weight (DW) using a rutin standard curve.

Total phenolics were quantified by the Folin–Ciocalteu method adapted from Irani et al. [26]. Dried leaf powder (0.4 g) was extracted with 5 mL absolute ethanol under sonication for 30 min. An aliquot (0.5 mL) of the supernatant was diluted with 9.5 mL distilled water, mixed with 1 mL 0.25 mol L^−1^ Folin–Ciocalteu reagent, and after 5 min, 2 mL 5% Na_2_CO_3_ was added. The mixture was incubated at 50 °C for 1.5 h, cooled to room temperature, and absorbance was measured at 760 nm. Total phenolic content was expressed as mg gallic acid equivalents g^−1^ dry weight (DW) using a gallic acid standard curve.

### 2.5. Identification and Quantification of Organic Acids

Organic-acid profiling was performed by high-performance liquid chromatography (HPLC) following Arnetoli et al. [27]. Instrumentation and conditions, Agilent 1260 HPLC; Waters XBridge C18 column (4.6 mm × 250 mm, 5 µm); mobile phase was 0.01 M KH_2_PO_4_ containing 10% methanol, pH 2.7 (adjusted with H_3_PO_4_); UV detection was set at 215 nm; flow rate was 0.5 mL min^−1^; column temperature 30 °C; injection volume was 20 µL. Sample preparation, Fresh lettuce (0.5 g) was ground in liquid nitrogen with 3 mL ultrapure water and transferred to a 15 mL tube with 5 mL additional water. The slurry was shaken in an 80 °C water bath for 1 h, cooled 30 min, and centrifuged at 8000 rpm (4 °C, 10 min). The supernatant (1.5 mL) was withdrawn, the pellet was re-extracted with an equal volume of water, and the combined supernatants (3 mL) were brought to 10 mL with ultrapure water. After 15 min sonication, the solution was filtered through 0.22 µm PVDF into a 1.5 mL amber vial. Quantification, External standards (malic and citric acids) were run under identical conditions. Peak area (X) versus concentration (Y) furnished regression equations and correlation coefficients (R^2^ ≥ 0.999). Malic and citric acid contents are expressed as mg·g^−1^ fresh weight (FW).

### 2.6. Root Architecture Analysis

Roots were gently rinsed with deionized water to remove adhering substrate, spread without overlap in a transparent tray, and scanned at 600 dpi with an MRS-9600TFU2L root scanner. Total root length, mean diameter, total volume, and surface area were computed from the digitized images with WinRHIZO Pro software (version 2025a, WinRHIZO, Shanghai, China).

### 2.7. Root Surface-Associated Microbiome Analysis

Loosely adhering soil was removed by gentle shaking. To collect the rhizoplane-associated microbial fraction (i.e., the root surface-associated community) Root systems were transferred to a sterile 50 mL tube containing 10 mL ice-cold 0.1 M potassium phosphate buffer (pH 8.0) and vortexed at maximum speed for 30 s; this wash step was repeated twice. Roots were then immersed in 20 mL fresh buffer and sonicated (160 W, 30 s on/30 s off, 10 min) to dislodge the rhizoplane fraction. The three wash solutions were pooled, vacuum filtered through a 0.22 µm polycarbonate membrane, and the filters were snap-frozen in liquid nitrogen and stored at −80 °C until shipment. DNA extraction, Illumina MiSeq paired-end sequencing (2 × 250 bp) of the V3–V4 region of the 16S rRNA gene, and bioinformatic analyses were performed by Majorbio Bio-Pharm Technology Co., Ltd. (Shanghai, China) on their cloud platform.

### 2.8. Determination of Soil Physicochemical Properties

Soil physicochemical properties were determined according to Chinese national standards. Specifically, soil organic matter was measured by the potassium dichromate spectrophotometric method [28]; available phosphorus was extracted with 0.5 M NaHCO_3_ and analyzed by the molybdenum-blue colorimetric procedure [29]; available potassium was extracted with 1 M ammonium acetate and determined by flame photometry [30]; bulk density and moisture content were obtained gravimetrically [31]; electrical conductivity and pH were measured potentio-metrically with glass and combination electrodes, respectively [32]; and the soil nitrogen uptake rate was estimated by the copperized-cadmium reduction diazotization-coupling colorimetric method [33]. At harvest, rhizosphere soil was collected for the determination of soil physicochemical properties. In addition, undisturbed soil samples were collected using a stainless-steel ring cutter with a known volume of 60 cm^3^. The fresh weight of each soil core was recorded immediately after sampling. The samples were then oven-dried at 110 °C to constant weight, and the dry weight was measured. Soil bulk density was calculated as the oven-dry mass of soil divided by the initial core volume (60 cm^3^) and expressed as g·cm^−3^. Gravimetric soil moisture content was calculated as the difference between fresh weight and dry weight divided by the dry weight and expressed as a percentage [34].

### 2.9. Determination of NR, GS, and GOGAT Activities

Enzyme activities were determined using kit-based assays. The nitrate reductase, glutamine synthetase, and glutamate synthase activity assay kits were obtained from ACMEC, Amole, and Aladdin, respectively.

### 2.10. Statistical Analysis

Data were initially processed using Microsoft Excel. Statistical analyses were performed using SPSS 22.0, and differences among treatments were first tested by one-way ANOVA. When the ANOVA was significant, Dunnett’s multiple-comparison test was used to compare each treatment with the control. Graphical visualization and supplementary analyses were conducted using GraphPad Prism 9.5.0. Results are presented as means ± standard error (SE), with three replicates per treatment (*n* = 3–5). Differences were considered statistically significant at * *p* < 0.05 and ** *p* < 0.01.

Related microbial analyses, including PCA, NMDS, ADONIS, and RDA, were performed on the Majorbio bioinformatics platform (Majorbio Cloud Platform, Shanghai, China). Specifically, PCA was conducted using Z-score normalization, and NMDS was based on the Bray–Curtis similarity matrix. Differences in community structure among treatment groups were assessed using ADONIS. For LEfSe analysis, the Kruskal–Wallis rank-sum test was first used to detect differences in species abundance among groups and to identify significantly differentially abundant taxa. Pairwise comparisons were then performed using the Wilcoxon rank-sum test, and linear discriminant analysis (LDA) was used to estimate the effect size of each differentially abundant feature.

## 3. Results

### 3.1. Growth Performance of L. sativa var. longifolia

**Figure 1:**
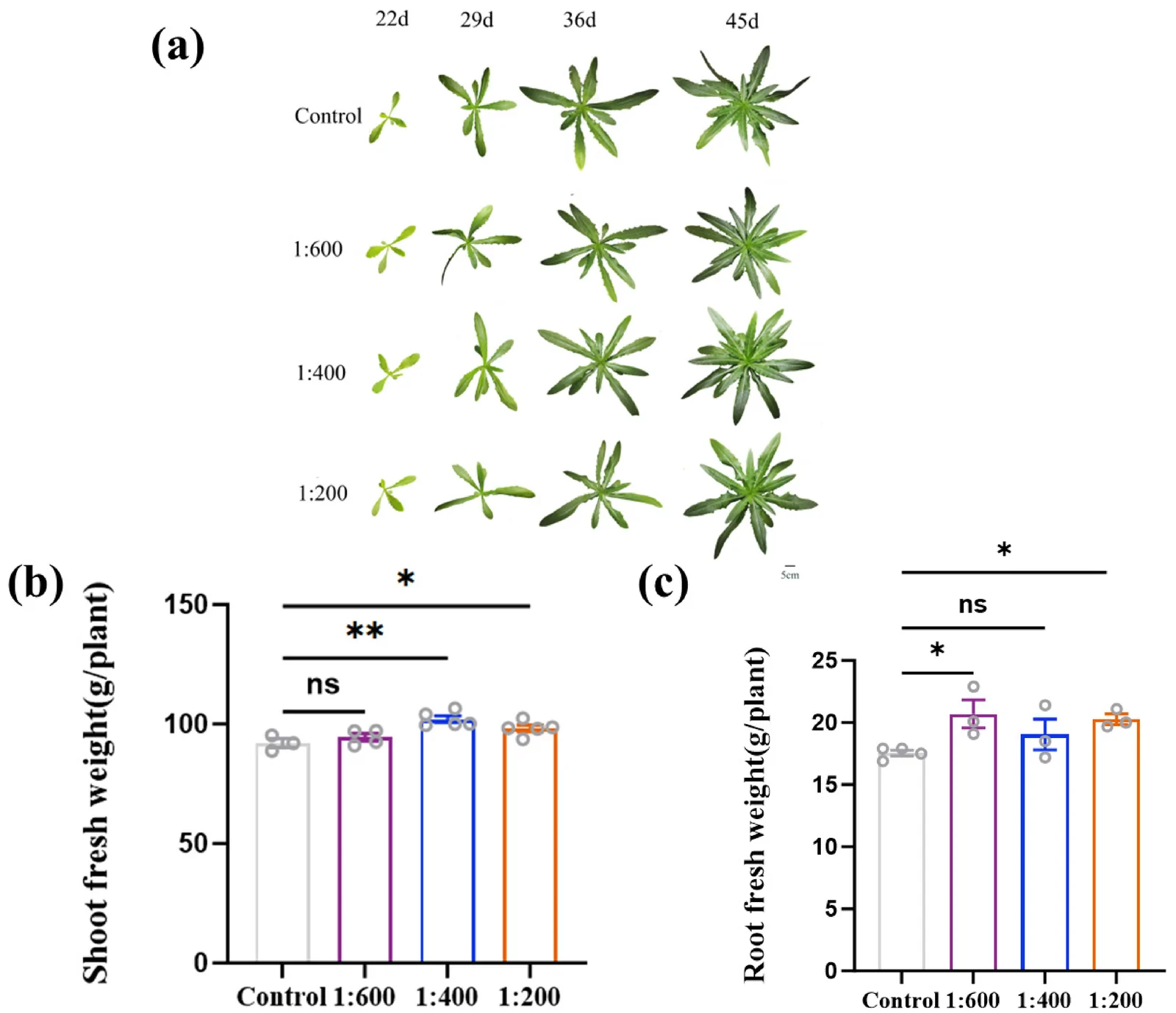
Effects of different concentrations of *U. lactuca* extract on lettuce growth (**a**) and fresh weight of above-ground (**b**) and below-ground (**c**) parts. Data are presented as mean ± SD (*n* = 3–5). Asterisks indicate significant differences (* *p* < 0.05, ** *p* < 0.01); “ns” indicates no significant difference.

Figure 1a illustrated the response of *L. sativa* var. *longifolia* shoot biomass to different dilutions of the *U. lactuca* extract. Among the three extract treatments, the 1:400 dilution resulted in the most pronounced increase in above-ground growth (*p* < 0.01). Although both shoot and root fresh weights values were numerically higher than those of the control in all extract-treated plants, significantly differences were treatment-dependent. Root fresh weight was significantly increased in 1:600 and 1:200 treatments, whereas shoot fresh weight was significantly increased in the 1:400 and 1:200 treatments (Figure 1b,c).

**Figure 2:**
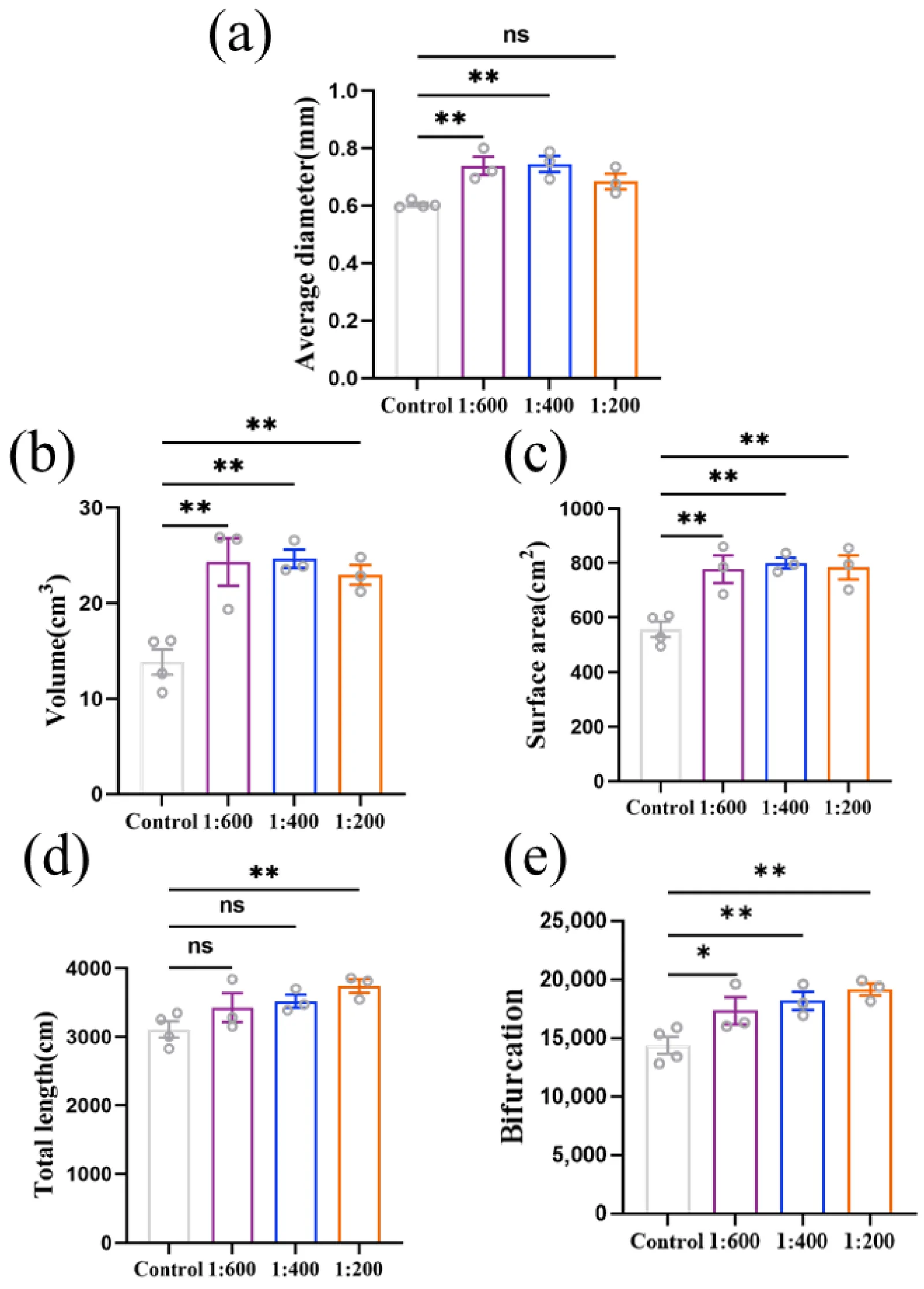
Histograms of root-related parameters: average diameter (**a**), volume (**b**), surface area (**c**), total length (**d**), and number of forks (**e**) for lettuce roots. Data are presented as mean ± SD (*n* = 3–5). Asterisks indicate significant differences (* *p* < 0.05, ** *p* < 0.01); “ns” indicates no significant difference.

Application of the *U. lactuca* extract increased mean root diameter, surface area, volume, total length and the number of root forks of *L. sativa* var. *longifolia*, but the magnitude of the response differed among concentrations. At 1:600 and 1:400 dilutions, mean root diameter was significantly higher than in control (Figure 2a). Surface area, volume and total bifurcation number were significantly higher than in the control under all three extract treatments (Figure 2b,c,e). Total root length was significantly increased only at 1:200 dilution, while no significant changes were observed at the 1:600 and 1:400 dilutions (Figure 2d).

### 3.2. Nutritional Quality of L. sativa var. longifolia

**Figure 3:**
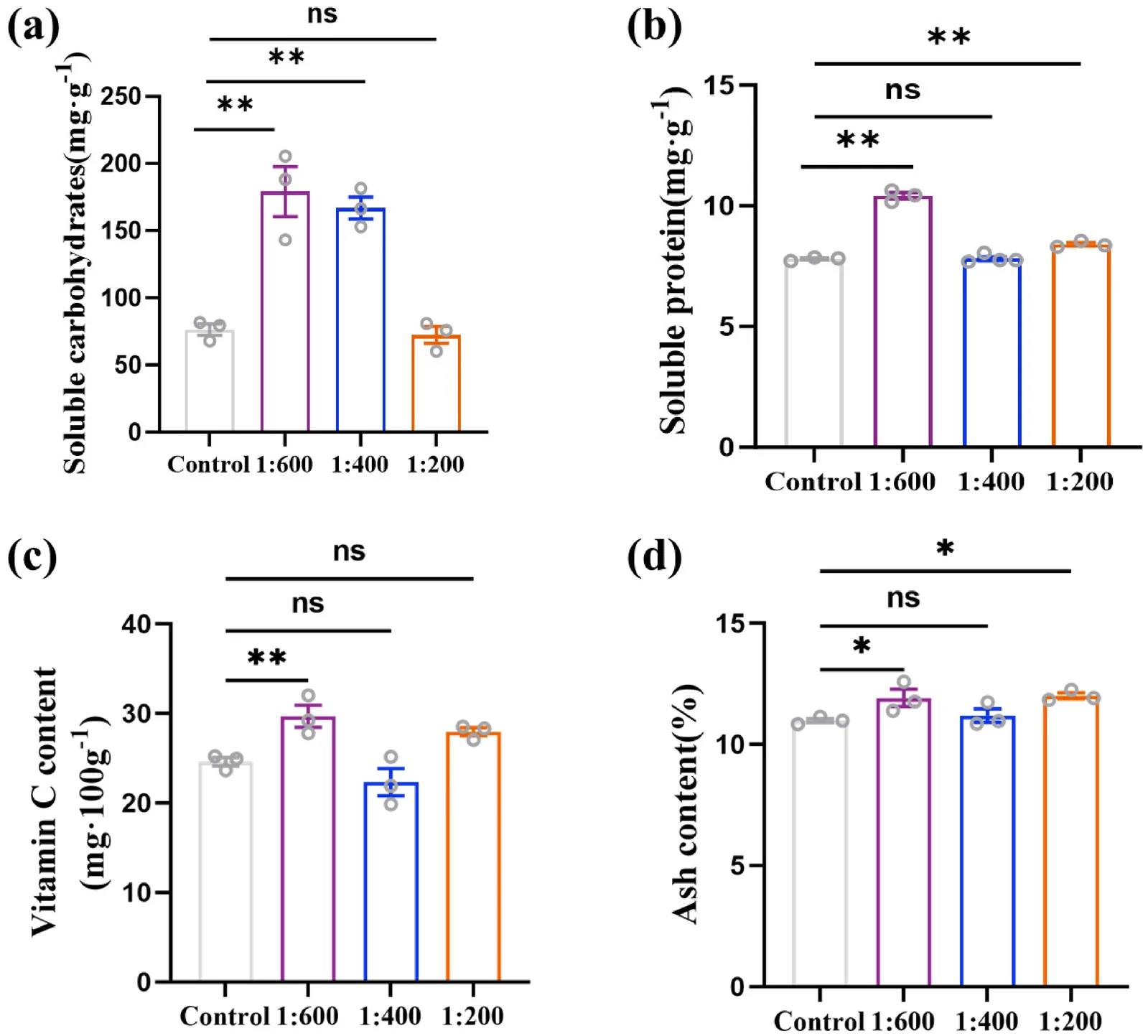
Histograms of lettuce nutritional indices: soluble sugar (**a**), soluble protein (**b**), vitamin C content (**c**), and ash content (**d**). Data are presented as mean ± SD (*n* = 3–5). Asterisks indicate significant differences (* *p* < 0.05, ** *p* < 0.01); “ns” indicates no significant difference.

As shown in Figure 3, foliar application of the *U. lactuca* extract improved the nutritional quality of *L. sativa* var. *longifolia*. Soluble sugar concentrations in the 1:600 and 1:400 treatments were significantly higher than in the control (*p* < 0.01), whereas the 1:200 treatment showed no significant difference (*p* > 0.05; Figure 3a). Soluble protein content was significantly increased in 1:600 (*p* < 0.01) and 1:200 (p < 0.01), but remained unchanged in the 1:400 group (*p* > 0.05; Figure 3b). Vitamin C content reached a maximum in the 1:600 treatment, increasing by 20.62% relative to the control (*p* < 0.01); conversely, the 1:400 treatment reduced vitamin C by 9.31% (Figure 3c). Ash content was also significantly higher in the 1:600 and 1:200 treatments, rising by 8.5% and 9.28%, respectively (*p* < 0.05; Figure 3d).

### 3.3. Carbon-Assimilation Capacity of L. sativa var. longifolia

**Figure 4:**
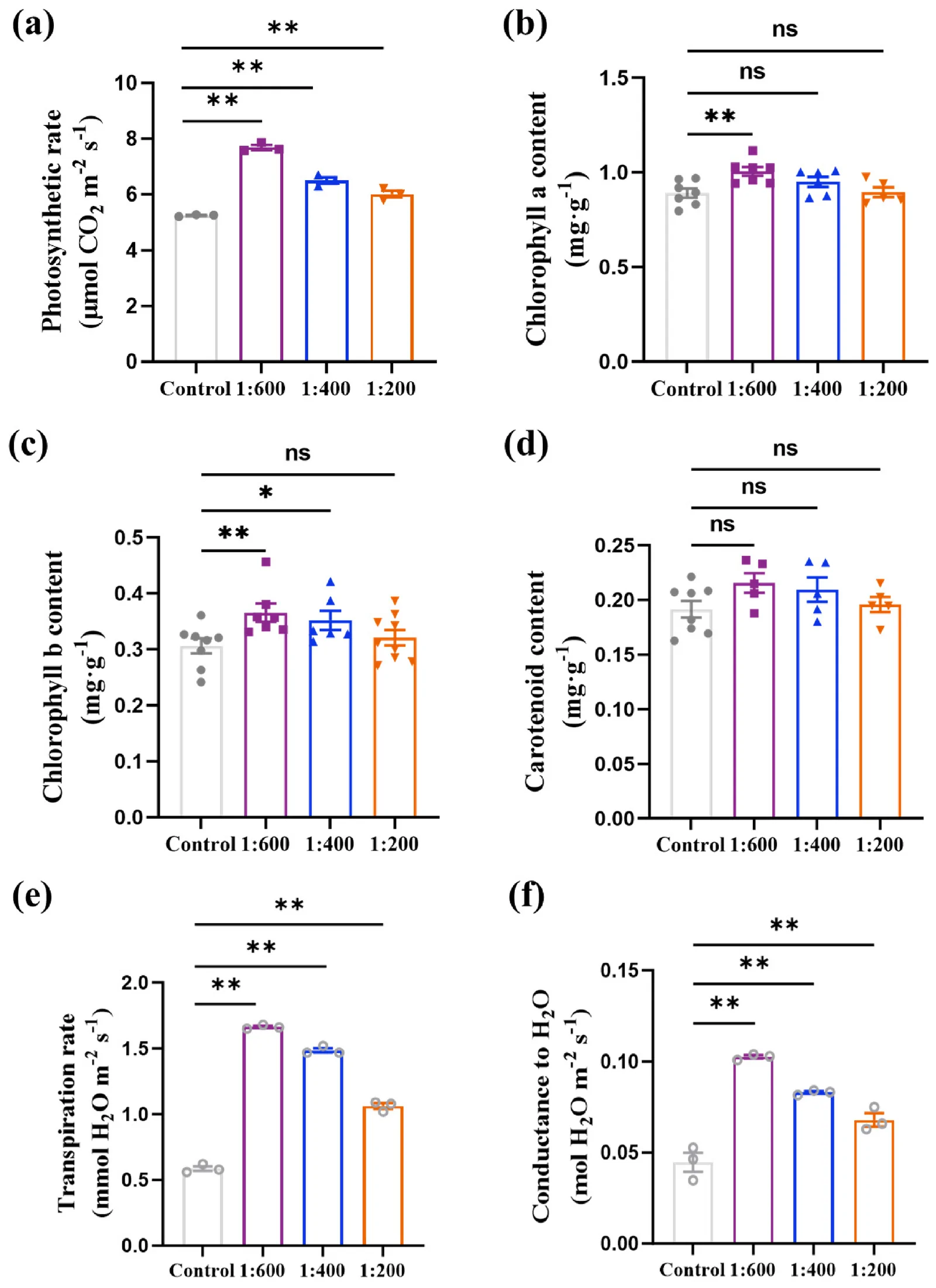
Effects of different concentrations of *U. lactuca* extract on net photosynthetic rate (**a**), photosynthetic pigments (**b**–**d**), transpiration rate (**e**), and stomatal conductance (**f**) in lettuce. Data are presented as mean ± SD (*n* = 3–5). Asterisks indicate significant differences (* *p* < 0.05, ** *p* < 0.01); “ns” indicates no significant difference.

As shown in Figure 4, net photosynthetic rate (Pn) in extract-treated lettuce exceeded that of the control and increased progressively with dilution ratio, peaking at 1:600 where Pn was 46.48% higher than the control (*p* < 0.01; Figure 4a). The same trend was observed for photosynthetic pigments: Chl a and Chl b contents reached maxima at 1:600, rising by 12.87% and 19.36% relative to the control (*p* < 0.01; Figure 4b,c), whereas carotenoid content remained unchanged (Figure 4d).

In addition, both stomatal conductance (Gs) and transpiration rate (Tr) of lettuce exceeded the control after *U. lactuca* extract application and increased with dilution ratio. Compared with the control, Tr rose by 183.52%, 153.41% and 81.25% under 1:600, 1:400 and 1:200 treatments, respectively, with the increase being highly significant (*p* < 0.01) (Figure 4e,f).

### 3.4. Changes in Nitrogen-Assimilation Activities of NR, GS and GOGAT

**Figure 5:**
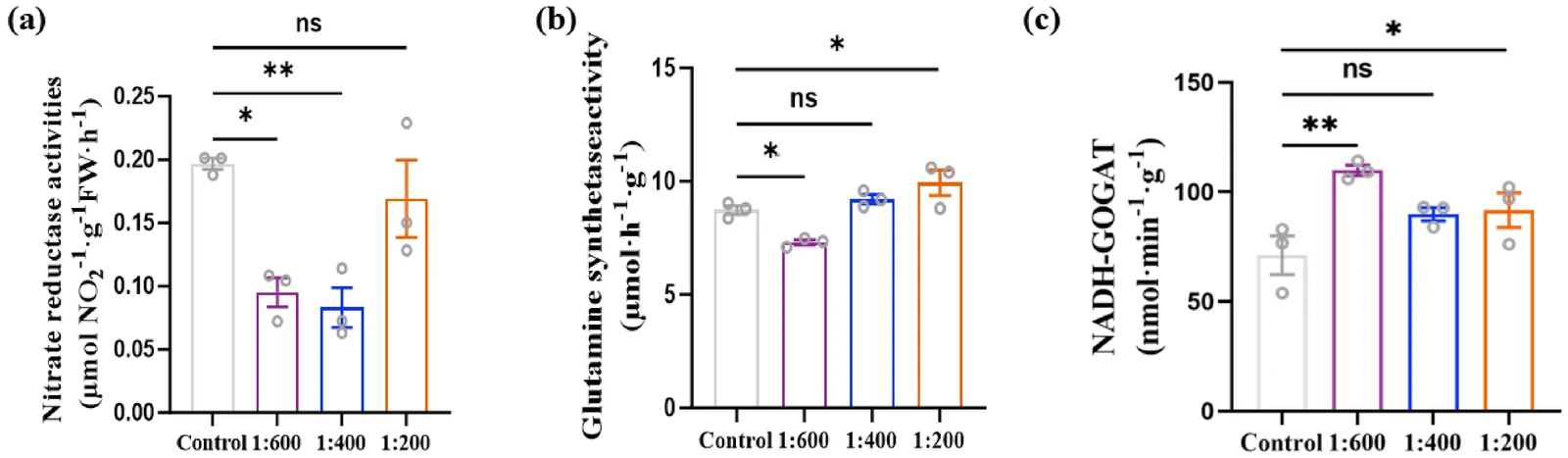
Effects of different concentrations of *U. lactuca* extract on nitrate reductase (NR) activity (**a**), glutamine synthetase (GS) activity (**b**), and NADH-dependent glutamate synthase (NADH-GOGAT) activity (**c**) in lettuce. Data are presented as mean ± SD (*n* = 3–5). Asterisks indicate significant differences (* *p* < 0.05, ** *p* < 0.01); “ns” indicates no significant difference.

As shown in Figure 5, relative to the control, nitrate reductase (NR) activity was significantly decreased in the 1:600 (*p* < 0.05) and 1:400 (*p* < 0.01) groups, but showed no significant change in the 1:200 group (Figure 5a). Glutamine synthetase (GS) activity was significantly decreased in the 1:600 group (*p* < 0.05) and significantly increased in the 1:200 group (*p* < 0.05), whereas no significant difference was observed in the 1:400 group (Figure 5b). Glutamate synthase (GOGAT) activity was significantly increased in the 1:600 (*p* < 0.01) and 1:200 (*p* < 0.05) groups, but did not differ significantly from the control in the 1:400 group (Figure 5c).

### 3.5. Secondary Metabolites of L. sativa var. longifolia

**Figure 6:**
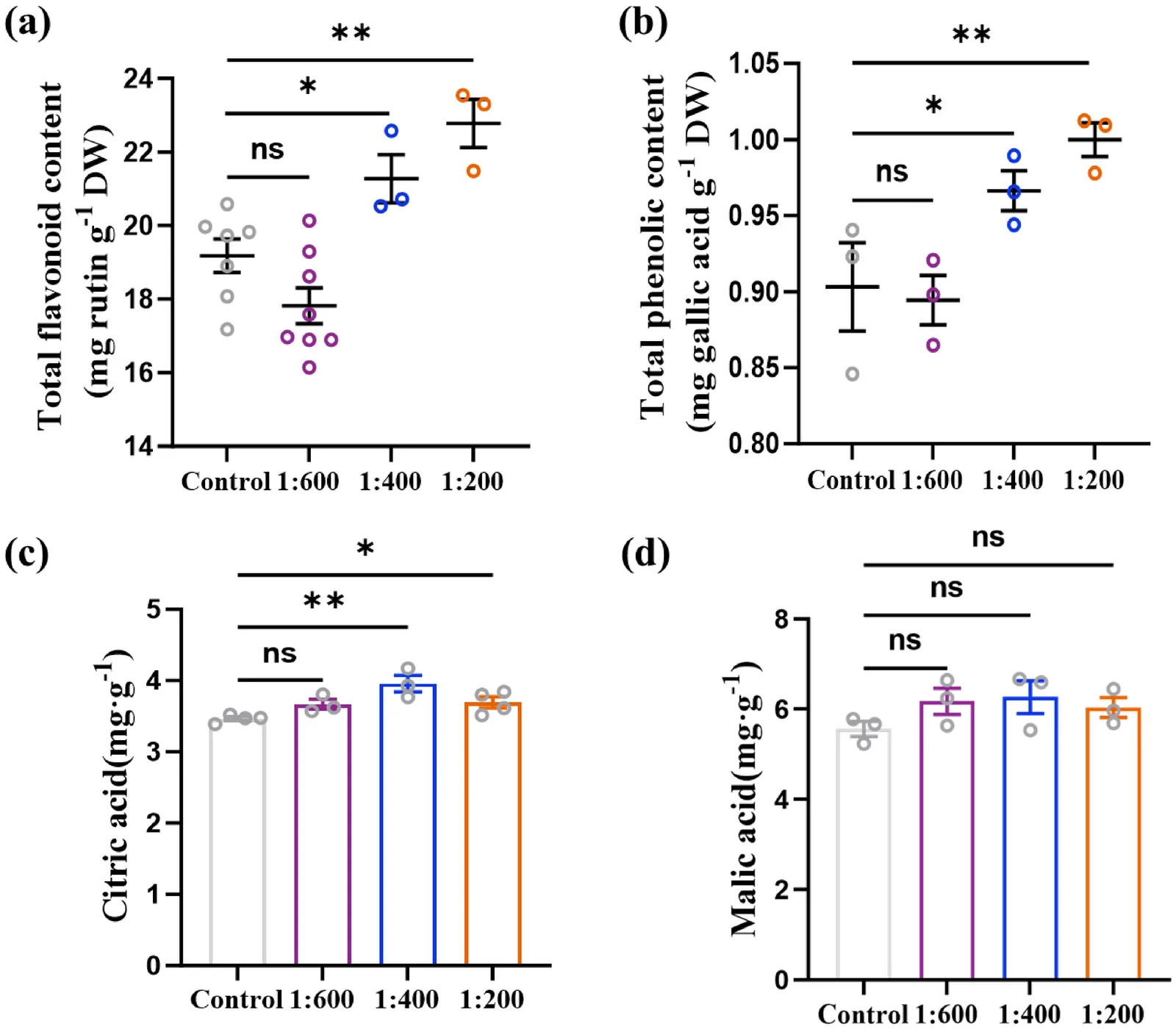
Bar charts of secondary metabolites in lettuce: total flavonoid content (TFC) (**a**), total phenolic content (TPC) (**b**), citric acid content (**c**), and malic acid content (**d**). Data are presented as mean ± SD (*n* = 3–5). Asterisks indicate significant differences (* *p* < 0.05, ** *p* < 0.01); “ns” indicates no significant difference.

As shown in Figure 6, total flavonoid and total phenolic contents in *L. sativa* var. *longifolia* increased gradually with the concentration of the *U. lactuca* extract. Plants treated with 1:400 and 1:200 dilutions exhibited significantly higher levels of both total flavonoids and total phenolics compared to the control (*p* < 0.05) (Figure 6a,b). The 1:400 dilution induced the most pronounced enhancement in citric acid content, increasing it by 14.15% relative to the control (*p* < 0.01) (Figure 6c). Although malic acid content was elevated under all extract treatments, none of the changes reached statistical significance compared with the control (Figure 6d).

### 3.6. Physicochemical Properties of Soil

**Figure 7:**
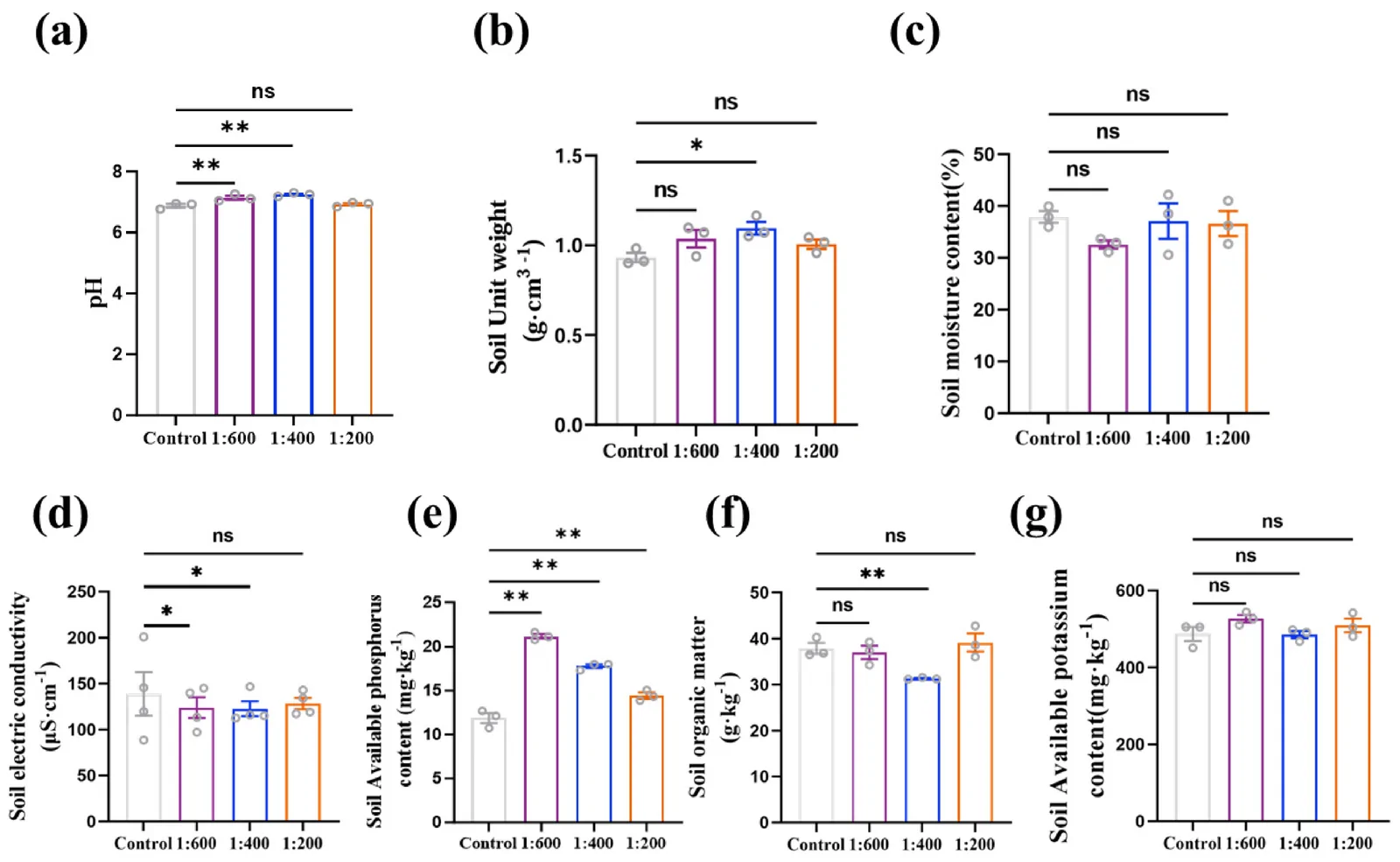
Effects of different concentrations of *U. lactuca* extract on soil physicochemical properties: pH (**a**), bulk density (**b**), soil moisture content (**c**), electrical conductivity (**d**), available phosphorus (**e**), soil organic matter (**f**), and available potassium content (**g**). Data are presented as mean ± SD (*n* = 3–5). Asterisks indicate significant differences (* *p* < 0.05, ** *p* < 0.01); “ns” indicates no significant difference.

The application of *U. lactuca* extract affected selected physicochemical properties of the soil. As shown in Figure 7a, soil pH remained within the neutral range under all treatments. Compared with the control, soil pH was significantly higher in the 1:600 treatment (*p* < 0.01) and highly significantly higher in the 1:400 treatment (*p* < 0.01), whereas no significant difference was observed in the 1:200 treatment.

Furthermore, as shown in Figure 7b, under the 1:400 concentration treatment, the soil bulk density significantly increased compared with the control (*p* < 0.05), whereas no significant differences were observed in the other two groups. Regarding soil water content, there were no significant differences among the treatment groups compared with the control (Figure 7c). The application of varying concentrations of *U. lactuca* extract resulted in an increase in soil available phosphorus content when compared to the control group, with the 1:600 treatment group exhibiting the most pronounced increase (*p* < 0.01) (Figure 7e). Conversely, no significant differences were observed in soil available potassium content across the treatments (Figure 7g). Interestingly, under the 1:400 dilution treatment, a decrease in organic matter content was noted (Figure 7f). Additionally, soil electrical conductivity decreased across all treatments relative to the control group (Figure 7d).

Soil moisture content and bulk density were also affected by the treatments (Figure S1). Soil moisture content was significantly lower in the 1:600 treatment than in the control, whereas no significant differences were observed between the control and the 1:400 or 1:200 treatments (Figure S1a). Soil bulk density was significantly higher in the 1:400 treatment than in the control, while the 1:600 and 1:200 treatments did not differ significantly from the control (Figure S1b).

### 3.7. Diversity and Richness Indices of the Soil Rhizobacterial Community

**Figure 8:**
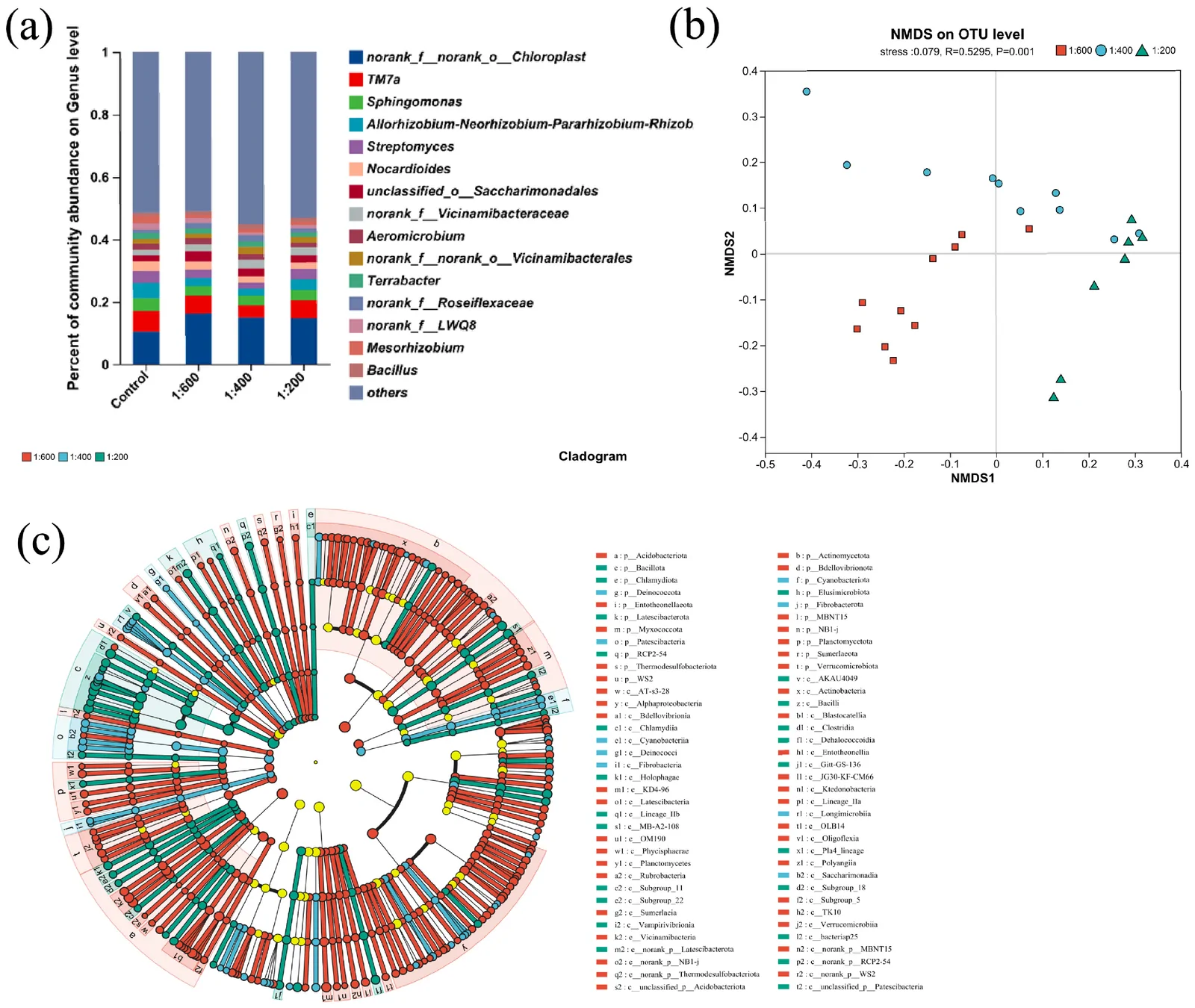
Variations in the composition of the root surface-associated microbial community among different treatment groups (**a**); non-metric multidimensional scaling (NMDS) ordination showing the differences in the structure of the root surface-associated microbial community among the three treatment groups (**b**); taxonomic cladogram generated by linear discriminant analysis (LDA) illustrating the dissimilarities in the root surface-associated microbial community among treatment groups (**c**).

As shown in Figure 8a, the application of *U. lactuca* extract altered the relative abundance of rhizobacterial communities at the genus level. The dominant genera across treatments included norank_f__norank_o__Chloroplast, TM7a, Sphingomonas, Allorhizobium–Neorhizobium–Pararhizobium, Streptomyces, Nocardioides, and several unclassified taxa. Overall, the relative abundance patterns of the dominant genera varied among treatments, indicating that the *U. lactuca* extract influenced the genus-level composition of the rhizosphere bacterial community. NMDS analysis at the OTU level showed a clear separation of rhizosphere bacterial communities among the 1:600, 1:400, and 1:200 treatments (Figure 8b). ADONIS analysis further confirmed that the community composition differed significantly among treatments (*p* = 0.001), indicating that different concentrations of *U. lactuca* extract altered the rhizobacterial community structure. The cladogram analysis identified 20 differential taxa among treatments, mainly at the phylum level (Figure 8c). Taxa enriched in the 1:600 treatment included Acidobacteriota, Actinomycetota, Bdellovibrionota, Entotheonellaeota, MBNT15, Myxococcota, NB1-j, Planctomycetota, Sumerlaeota, Thermodesulfobacteriota, Verrucomicrobiota, and WS2. Cyanobacteriota, Fibrobacterota, and Patescibacteria were enriched in the 1:400 treatment, whereas Bacillota, Chlamydiota, Elusimicrobiota, Latescibacterota, and RCP2-54 were enriched in the 1:200 treatment.

**Figure 9:**
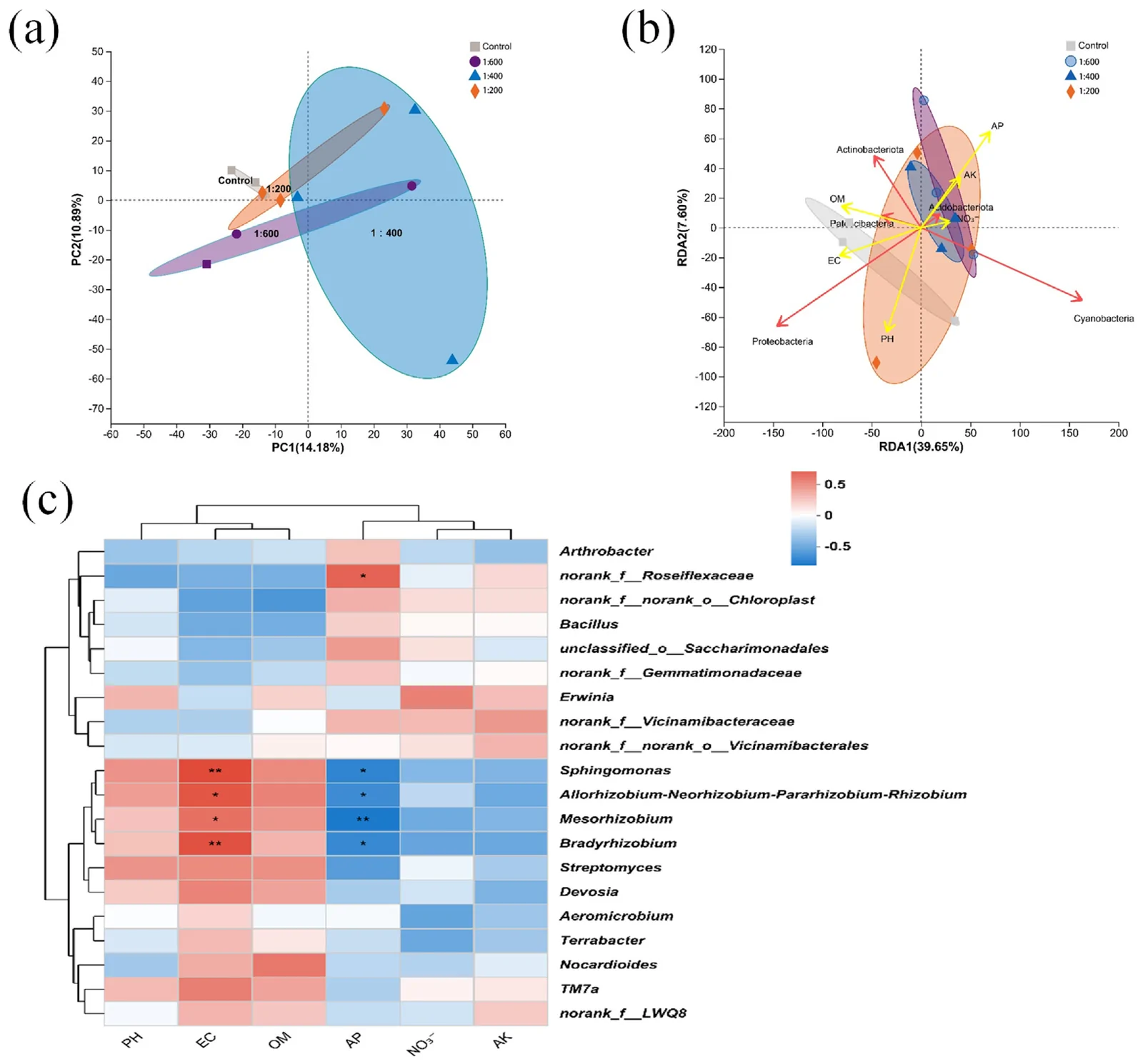
Effects of different concentrations of *U. lactuca* extract on rhizoplane microorganisms: PCA plot of rhizosphere soil microbial communities (**a**), Redundancy analysis (RDA) showing associations between substrate physicochemical properties and the rhizoplane-associated bacterial community composition. (**b**), and hierarchical clustering heatmap of rhizosphere soil microorganisms (**c**) Data are presented as mean ± SD (*n* = 3–5). Asterisks indicate significant differences (* *p* < 0.05, ** *p* < 0.01).

Principal coordinate analysis (PCoA) showed a separation of rhizosphere bacterial communities among the control, 1:600, 1:400, and 1:200 treatments (Figure 9a), indicating that *U. lactuca* extract application altered the overall bacterial community structure. Redundancy analysis (RDA) suggested that soil physicochemical properties were associated with variation in the rhizoplane-associated bacterial community (Figure 9c). Among the measured variables, pH and available phosphorus (AP) showed relatively strong associations with the ordination pattern. Proteobacteria was oriented in a direction similar to pH and opposite to AP, suggesting a positive association with pH and a negative association with AP. In contrast, Cyanobacteria, Patescibacteria, Actinobacteriota, and Acidobacteriota were oriented more closely with AP than with pH, indicating a different association pattern. These results suggest that variation in substrate properties was associated with shifts in the composition of the root surface-associated bacterial community. The correlation heatmap further showed distinct associations between dominant genera and soil properties (Figure 9c). Sphingomonas, Allorhizobium–Neorhizobium–Pararhizobium–Rhizobium, Mesorhizobium, Bradyrhizobium, Streptomyces, and Devosia were positively correlated with pH, EC, and OM, but negatively correlated with AP. In contrast, Arthrobacter, norank_f__Roseiflexaceae, norank_f__norank_o__Chloroplast, Bacillus, unclassified_o__Saccharimonadales, and norank_f__Gemmatimonadaceae showed the opposite pattern. Other genera, including Aeromicrobium, Terrabacter, Nocardioides, TM7a, and norank_f__LWQ8, also exhibited variable relationships with the measured soil properties.

## 4. Discussion

Our results indicated that foliar application of algal extract improved photosynthetic performance, as evidenced by the increase in net photosynthetic rate (Pn), and promoted plant growth, as reflected by higher shoot and root fresh weight in lettuce. In contrast, the responses of photosynthetic pigments were treatment-dependent rather than consistent across all dilutions: chlorophyll a increased significantly under the 1:600 treatment, chlorophyll b increased significantly under the 1:600 and 1:400 treatments, and carotenoid content was not significantly affected. Yang et al. [35] reported that a seaweed-based biostimulant increased Pn, stomatal conductance and chlorophyll content in sugar beet, while Chaudhary et al. [36] found that similar extracts were associated with enhanced photosynthesis in *Crocus sativus*, including higher Chl a, Chl b and carotenoid contents. Consistently with these reports, extract-treated lettuce in the present study exhibited higher Pn and pigment contents than the control, and the 1:400 dilution produced the greatest increase in shoot fresh weight. These responses were possibly related to bioactive compounds reported in algal extracts, such as phytohormone-like substances (auxins, cytokinins, gibberellins) and multifunctional metabolites (polysaccharides, polyphenols, flavonoids) which may stimulate chloroplast biogenesis and suppress chlorophyll degradation [10,37]. However, this interpretation remains speculative because the extract used in the present study was not chemically characterized. Moreover, these compounds could modulate endogenous signaling cascades [38], thereby improving light-energy conversion efficiency. Comparable findings by Shibaeva et al. [39] revealed that medium-concentration seaweed extracts markedly promoted biomass in wheat (*Triticum aestivum* L.) and cucumber (*Cucumis sativus* L.) seedlings, indicating that growth enhancement through optimized seaweed extracts was a general phenomenon across crops and vegetables.

The different responses observed among the three dilutions suggest a concentration-dependent mode of action of the *U. lactuca* extract. A plausible explanation is that the extract contains bioactive compounds with hormone-like and biostimulatory properties, such as auxin- and cytokinin-related substances, together with polysaccharides, phenolics, amino acids, and mineral nutrients (Table S3) [40,41,42,43]. At relatively low concentrations, these compounds may act mainly as signaling molecules, improving metabolic coordination and favoring the accumulation of quality-related constituents, which may help explain the stronger effect of the 1:600 dilution on nutritional traits. At an intermediate concentration, the balance between signaling stimulation and nutritional support may be more suitable for promoting cell division, root activity, assimilate production, and biomass accumulation [44], which is consistent with the stronger growth-promoting effect observed under the 1:400 treatment. By contrast, the reduced effect at the 1:200 dilution may indicate that excessive exogenous inputs weakened the biostimulatory response, possibly through osmotic effects, metabolic burden, or disturbance of endogenous hormonal homeostasis.

In terms of nutritional traits, algal extract increased soluble sugar, soluble protein, vitamin C, and ash contents, with the 1:600 dilution showing the strongest overall effect on these parameters. The extract also affected several secondary metabolites, although the responses depended on dilution level [45]. Citric acid increased significantly only under the 1:400 treatment, whereas malic acid showed no significant change among treatments. Likewise, total phenolic and total flavonoid contents were higher in the 1:400 and 1:200 treatments, but not in the 1:600 treatment. These results indicate that different quality-related traits responded differently to extract concentration. Such divergence may reflect different sensitivity thresholds of primary and secondary metabolic processes to the bioactive substances supplied by the extract. In this study, the 1:600 dilution appeared to be more favorable for the accumulation of soluble sugar, soluble protein, vitamin C, and ash, suggesting that lower extract doses may preferentially support metabolic regulation associated with nutritional quality, whereas higher concentrations did not further enhance these traits. Any implications for antioxidant capacity, vacuolar pH, pathogen suppression, or stress tolerance were not directly evaluated in this study and therefore remain speculative. It has been mentioned in other studies, the marked increases in total phenolics and flavonoids implied elevated antioxidant activity, probably linked to up-regulation of glutathione reductase and ascorbate peroxidase [46], which further strengthened stress tolerance. Comparable improvements in nutritional quality have been reported by Ali et al. [47] in tomato (*Solanum lycopersicum* L.) and pepper (*Capsicum annuum* L.) treated with seaweed extracts.

Foliar application of the algal extract also modified root architecture, significantly affecting mean diameter, total length, surface area, and fork number. Root proliferation was closely associated with available soil phosphorus; elevated Olsen-P enhanced lateral root initiation and branching intensity [48]. Consistent with these findings, the present study showed that extract treatments markedly increased soil AP relative to the control, thereby stimulating root development, improving stress tolerance and ultimately raising both biomass and quality of *L. sativa* var. *longifolia*. The concurrent reduction in soil bulk density improved aeration, creating a more favorable environment for root expansion.

These coordinated changes suggest that the effects of the algal extract were not restricted to leaf physiology alone, but may have extended to a broader soil–root interaction process. In particular, changes in soil physical and chemical properties may have improved the rhizosphere habitat, thereby facilitating root proliferation and creating conditions for subsequent shifts in microbial assembly [49].

Soil amelioration inevitably altered the resident microbiota, whose composition and diversity served as key indicators of ecological function [50]. Within the soil microbiome, Proteobacteria and Cyanobacteria contribute to nitrogen fixation [51], while Acidobacteria play a role in carbon cycling and nitrification [52]. Members of Patescibacteria are believed to promote root development [53]. Additionally, the rhizosphere microbiota establishes an intimate interaction with plants, directly shaping their growth, development, and resistance to abiotic stresses [54]. The algal extract provided readily available organic substrates, effectively stimulating microbial proliferation.

At the phylum level, extract application reduced the relative abundance of Proteobacteria in all treated soils and markedly decreased Patescibacteria at 1:400, while increasing the proportion of Cyanobacteria, particularly at 1:600. This apparent enrichment of Cyanobacteria should, however, be interpreted with caution, as the dominant lineage was assigned to the Chloroplast order rather than to a cyanobacterial class, indicating that the signal likely reflects plant-derived sequences rather than a true increase in free-living cyanobacteria. Therefore, these data should not be taken as direct evidence of enhanced nitrogen fixation. In contrast, the reduction in Proteobacteria suggests that the extract reshaped rhizosphere bacterial community structure. Given the ecological importance of many proteobacterial taxa in nutrient turnover and organic matter transformation, such changes may be relevant to rhizosphere processes [55], although targeted functional analyses would be required to confirm this.Compared with the control, extract application altered soil pH and rhizosphere bacterial community composition. However, the present data do not support a direct mechanistic link between the observed changes in Proteobacteria and soil pH variation. Therefore, the pH response should not be attributed to a specific microbial pathway on the basis of the current results alone. More generally, shifts in Proteobacteria may indicate that the extract influenced rhizosphere nutrient conditions and microbial assembly, but the functional consequences of these taxonomic changes require further validation.

Notably, the increase in AP may have enhanced nutrient availability and indirectly influenced rhizosphere microbial structure [56]. The relative abundances of Saccharimonadales and Vicinamibacteraceae showed treatment-dependent variation, with the 1:600 dilution exhibiting the most pronounced response in Figure 8a; however, a consistent increase across all extract treatments was not clearly supported. Similarly, although changes in Vicinamibacteraceae occurred together with changes in soil AP, the present study does not provide direct evidence that this taxon was responsible for phosphate solubilization. Thus, the observed association between microbial shifts and AP should be interpreted cautiously. These results suggest that algal extract application may influence phosphorus-related microbial assemblages, but the specific functional roles of these taxa remain to be verified. By contrast, the relative abundances of Sphingomonas and the Allorhizobium–Neorhizobium–Pararhizobium–Rhizobium clade declined under algal extract application, indicating that the extract may have reshaped the rhizosphere microbial network and altered the competitive balance among bacterial groups.

Changes in soil AP were also accompanied by variation in the abundance of several other bacterial taxa, including groups affiliated with Acidobacteriota. However, the present data do not support a direct causal pathway linking AP, Proteobacteria, pH, and the enrichment of Acidobacteriota. We therefore interpret these patterns as correlations rather than confirmed mechanisms. Overall, the co-variation among AP, pH, and bacterial taxa suggests that extract application modified the rhizosphere environment, but ecological functions cannot be inferred from taxonomic composition alone.

PCA at the phylum level revealed that the microbial communities of the treated groups were separated from the control, with the 1:400 treatment appearing farther from the control than the 1:600 treatment. RDA further suggested the substrate pH and AP were the physicochemical variables most strongly associated with variation in the rhizoplane-associated bacterial community. pH was positively associated with Proteobacteria and negatively associated with Actinobacteriota, Patescibacteria, and Acidobacteriota, whereas AP showed the opposite trend. Relative to the control, AP was significantly elevated in all extract treatments and this pattern was accompanied by a higher relative abundance of Actinobacteriota, a group widely recognized for its role in organic matter turnover and the maintenance of soil ecosystem functioning [57]. These findings suggest that algal extract application may influence lettuce performance partly through linked changes in substrate properties and the root surface-associated microbiome. However, because the physicochemical variables were measured in the cultivation substrate whereas the bacterial community was characterized from the rhizoplane-associated fraction, these relationships should be interpreted as cross-compartment associations rather than direct evidence of causal interactions. Further work is needed to clarify the functional mechanisms involved.

Taken together, the present results support a possible cascade pathway of “soil property regulation-microbial community remodeling-plant growth response.” Specifically, algal extract application was associated with changes in soil pH, bulk density, and available phosphorus, which may have modified the rhizosphere microenvironment and nutrient availability. These soil changes were accompanied by shifts in bacterial diversity and community composition, suggesting that the extract indirectly affected microbial assembly through altered habitat conditions and resource status. The reshaped rhizosphere environment may in turn have contributed to improved root development, nutrient acquisition, and physiological performance, ultimately leading to enhanced lettuce biomass and quality. It should be emphasized, however, that this pathway is inferred from coordinated patterns among soil, microbial, and plant variables rather than from direct causal evidence, and therefore requires confirmation by targeted functional and mechanistic studies.

In summary, foliar application of the algal extract was associated with improved lettuce growth and quality under the conditions of this study. Among the tested dilutions, the 1:400 dilution produced the greatest increase in shoot fresh weight, whereas the 1:600 dilution resulted in the highest soluble sugar, soluble protein, vitamin C, and ash contents, indicating a stronger effect on nutritional quality. The beneficial effects observed at appropriate concentrations were accompanied by higher net photosynthetic rate, photosynthetic pigment content, stomatal conductance, and changes in nitrogen-assimilation-related enzyme activities, suggesting that these physiological responses may have contributed to the growth improvement. In addition, the increases in total flavonoids, phenolics, and organic acids indicate that the extract may also influence secondary metabolism and quality-related traits. Changes in soil pH, bulk density, available phosphorus, and rhizosphere microbial diversity were also observed after extract application, suggesting a potential effect on the soil–plant–microbiome system. However, because the chemical composition of the extract and the underlying regulatory mechanisms were not directly characterized in this study, these interpretations should be considered preliminary. Further studies are needed to identify the active constituents of the extract and clarify the mechanisms responsible for the observed responses.

## 5. Conclusions

This study indicated that substances extracted from *U. lactuca*, an epiphytic green alga associated with cultivated *S. fusiforme*, had the potential to serve as marine-derived biostimulants in lettuce production. The effects were concentration-dependent: the 1:400 dilution was more effective in promoting shoot biomass, whereas the 1:600 dilution showed greater benefits for nutritional quality, significantly increasing soluble sugar, soluble protein, and vitamin C contents. Extract application was also associated with changes in selected soil properties and rhizosphere bacterial community composition, suggesting a potential influence on both plant performance and the rhizosphere environment.

From a practical perspective, the 1:400 dilution may be more suitable when higher biomass is the primary production goal, whereas the 1:600 dilution may be preferable when improved quality is targeted, particularly for high-value vegetable markets. Overall, epiphytic *Ulva* biomass represents a promising sustainable resource for agricultural use. However, because this study was conducted under controlled indoor conditions, further field validation, mechanistic investigation, and economic assessment are needed before large-scale application.

## Data Availability

The original contributions presented in this study are included in the article/supplementary material. Further inquiries can be directed to the corresponding author(s).

## Supplementary Materials

The following supporting information can be downloaded at https://www.mdpi.com/article/10.3390/biology15211936/s1, Figure S1: Microbial alpha diversity analysis; Table S1: Changes in epiphytic algae species and biomass (g·m^−2^) in a growth cycle of *Sargassum fusiforme*; Table S2: Nutritional quality of different *Ulva* species; Table S3: The bioactive components of *Ulva lactuca*.
