# Supplementary material for: Ulva lactuca Extract Increases the Growth of Lactuca sativa var. longifolia by Regulating Physiological States and Soil Microorganisms
Source: Biology (Basel). 2026 Oct 25;15(21):1936. doi: 10.3390/biology15211936 (PMC13761509; doi:10.3390/biology15211936)
Supplement: Supplementary file 1 [file biology-15-01936-s001.zip › biology-4622538-supplementary.pdf]

Supplementary information

Ulva lactuca extract increases the growth of *Lactuca sativa* var. *longifolia* by regulating physiological state and soil microorganisms

Authors

Dongshun Fang[1,2]; Mengying Yang[2]; Qiyun Xie[2]; Zihan Zhang[2]; Mingjiang Wu[1,3]; Haibin Tong[1,3]; Binbin Chen[1,3]*

Affiliations

[1] National and Local Joint Engineering Research Center of Ecological Treatment Technology for Urban Water Pollution, Wenzhou University, Wenzhou 325035, China;

[2] College of Life and Environmental Science, Wenzhou University, Wenzhou 325035, China;

[3] Zhejiang Provincial Key Laboratory of Water Ecological Environment Treatment and Resource Protection, Wenzhou University, Wenzhou 325035, China.

***Corresponding Author**

B Chen, Wenzhou University, Chashan University Town, Wenzhou 325035, China, Email: binbch@163.com.

Supplementary Table S1: Changes of epiphytic algae species and biomass (g·m^{-2}) in a growth cycle of *Sargassum fusiforme*.

Species	Nov	Dec	Jan	Mar	Mar*	Apr	Apr*
**Cyanophyta**							
*Cladophora fascicularis***	0.00	0.00	0.00	0.00	20.67	0.00	0.00
*Cladophora stimpsonii***	0.00	0.00	0.00	0.00	125.67	15.17	0.00
*Cladophora hutchinsioides***	0.66	7.02	0.00	0.00	0.00	0.00	0.00
Chaetomorpha spiralis	0.00	0.00	0.00	0.00	0.35	0.00	0.00
Bryopsis sp.	0.00	5.52	0.00	0.00	294.33	147.00	856.50
Codium fragile	0.00	3.13	0.00	0.00	338.00	0.00	0.00
Ulva lactuca	512.33	390.50	156.00	70.33	0.00	2.33	72.67
**Rhodophyta**							
*Acrosorium venulosum***	0.00	0.00	0.00	0.00	0.00	0.00	0.00
Ceramium sp.**	15.74	30.98	152.17	451.67	288.33	72.16	3.00
Amphiroa ephedraea	0.00	0.00	0.00	0.00	35.10	0.00	0.00
Grateloupia sp.	0.00	0.00	67.33	16.31	1900.61	597.34	612.17
*Halymenia floresia***	0.00	0.00	0.00	0.00	0.00	0.00	17.83
**Phaeophyta**							
*Petalonia fascia***	0.00	0.00	40.50	0.73	43.33	20.33	0.00
*Sargassum hornei***	0.00	0.00	0.00	0.00	0.00	0.00	0.00
**Number**	3	6	4	4	12	6	6
**Biomass**	528.74	437.35	416.00	539.05	3104.73	854.33	1570.17

* indicates algae attached to the main rope; ** indicates species not previously recorded in the literature.

Supplementary Table S2: Nutritional quality of different *Ulva* species.

Nutritional value of *Ulva rigida*[11]		Nutritional value of *Ulva* sp.[12]	
Total Content (g/100 g dw)		g/100 g DM	
Total Carbohydrates	58.1±0.7	Arginine	4.74
Fiber		Histidine	1.29
Total	36.6±1.5	Leucine	5.87
Soluble	17.7±1.1	Lysine	3.89
Insoluble	18.9±0.8	Insoluble	3.61
Ash	31.7±0.6	Ash	48.2±0.18
Minerals (mg/ 100 g dw)		Macrominerals (g/100 g DM)	
Na	242.0±233.8	Na	4.95 ± 0.10
K	2466.6±65.2	K	0.23 ± 0.00
Ca	414.3±33.8	Ca	6.56 ± 0.17
Mg	3758.6±430.3	Mg	2.11 ± 0.03
Fe	110.2±22.6	Microminerals (mg/kg DM)	
Mn	6.84±1.05	Fe	5079±305
Cu	3.27±0.26	Mn	1675±101
Zn	3.27±0.26	Cu	7.76±0.47
Ni	1.07±0.09	Zn	23.2±1.39
Protein	9.3±0.5	P	2300±0.00
Lipid	0.9±0.1	I	47.1±1.88
Caloric content(kcal/100 g dw)	167.3		

Supplementary Table S3: The bioactive components of *Ulva lactuca*

species	Polyphenols / Flavonoids	Phytohormones	Amino Acids	Other Key Bioactive Compounds
Ulva lactuca	Polyphenols: 65.640 ± 5.173 mg/g DW<br>Flavonoids: 45.760 ± 1.609 mg/g DW [40]	IAA: 0.577 ± 0.004 mg/g DW [40]	Free amino acids: 3.369 ± 0.002 mg/g DW [40]	Glycine betaine: 8.033 mg/g DW<br>Soluble sugars: 0.296 mg glucose/g DW [41]
	Polyphenols: 112.640 mg GAE/g DW<br>Flavonoids: 4.870 mg quercetin/g DW [41]			Total sugar: ~52.61%<br>Uronic acid: Present<br>Sulfate group: Present<br>Monosaccharide composition: Rhamnose 45.33%, Glucuronic acid 15.50%, Glucose 18.97%, Xylose 20.29%<br>Molecular weight: 891.25 kDa [43]
	Phenolics: 7 types detected by LC-MS/MS (gallic acid, 4-hydroxybenzoic acid, caffeic acid, vanillic acid, syringic acid, quercetin, kaempferol) [42].			

Supplementary Figure S1: Microbial alpha diversity analysis

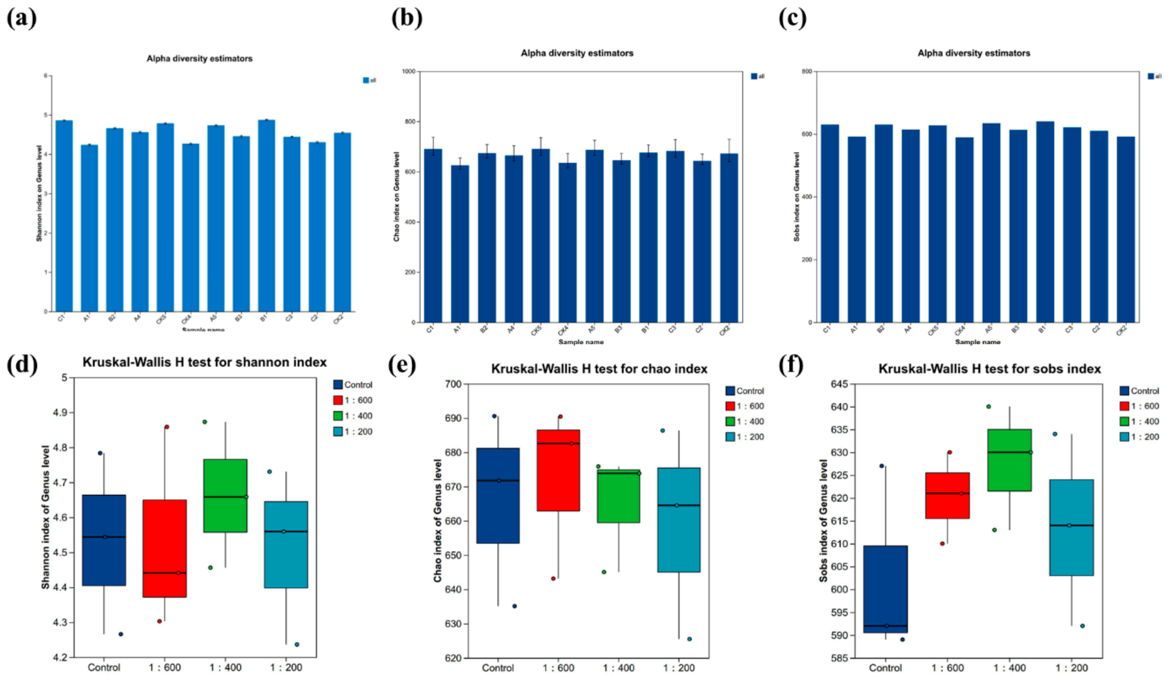

Figures a, b, and c present the microbial Shannon, Chao1, and Sobs indices, respectively. Figures d, e, and f show the between-group differences for each respective analysis.
